# Comparative Physiological and Transcriptomic Analyses of Improved Heat Stress Tolerance in Celery (*Apium Graveolens* L.) Caused by Exogenous Melatonin

**DOI:** 10.3390/ijms231911382

**Published:** 2022-09-27

**Authors:** Mengyao Li, Jin Zhou, Jiageng Du, Xiaoyan Li, Yue Sun, Zhuo Wang, Yuanxiu Lin, Yunting Zhang, Yan Wang, Wen He, Xiaorong Wang, Qing Chen, Yong Zhang, Ya Luo, Haoru Tang

**Affiliations:** 1 College of Horticulture, Sichuan Agricultural University, Chengdu 611130, China; 2 Institute of Pomology & Olericulture, Sichuan Agricultural University, Chengdu 611130, China

**Keywords:** antioxidant capacity, *Apium graveolens* L., exogenous melatonin, heat stress, transcriptomic analyses

## Abstract

Melatonin (MT) is crucial in plant growth, development, and response to stress. Celery is a vegetable that grows in a cool climate, and a hot climate can deteriorate its growth, yield, and quality. This study investigates the effect of exogenous melatonin on celery physiology. Transcriptional levels were analyzed by spraying celery with exogenous MT before exposing it to high temperatures. The regulatory mechanism of exogenous MT-mediated heat tolerance was examined. The results show that the exogenous MT reduced the thermal damage state of celery seedlings, as well as the malondialdehyde (MDA) content and relative conductivity (REC), increasing the oxidase activity, the osmotic regulatory substances, and chlorophyll, enhancing the leaf transpiration and the light energy utilization efficiency. We examined the mechanism of exogenous MT in mitigating high-temperature damage using the transcriptome sequencing method. A total of 134 genes were expressed differently at high temperature in the celery treated with MT compared with the untreated celery. Functional annotation analysis revealed that the differentially expressed genes were abundant in the “pyruvate metabolism” pathway and the “peroxidase activity” pathway. According to the pathway-based gene expression analysis, exogenous MT can inhibit the upregulation of pyruvate synthesis genes and the downregulation of pyruvate consumption genes, preventing the accumulated pyruvate from rapidly upregulating the expression of peroxidase genes, and thereby enhancing peroxidase activity. RT-qPCR verification showed a rising encoding peroxidase gene expression under MT treatment. The gene expression pattern involved in pyruvate anabolism and metabolism agreed with the abundant transcriptome expression, validating the physiological index results. These results indicate that the application of exogenous MT to celery significantly enhances the ability of plant to remove reactive oxygen species (ROS) in response to heat stress, thereby improving the ability of plant to resist heat stress. The results of this study provide a theoretical basis for the use of MT to alleviate the damage caused by heat stress in plant growth and development.

## 1. Introduction

The greenhouse effect has led to global warming, resulting in extremely high-temperature weather that challenges plant cultivation [1]. Heat stress can adversely affect plant cultivation, yield and distribution range, altering the morphology of plant leaves and the submicroscopic structure in cells [2], increasing the protoplasm membrane permeability and the tissue leaching solution conductivity [3]. Heat stress also disrupts the intracellular oxygen-free radical balance, leading to oxidative damage in plants [4]. In addition, the maximum light energy conversion efficiency of plants (Fv/Fm), their light energy capture efficiency (Fv’/Fm’), and their photosynthetic system II (PS II) electron transfer efficiency are reduced [5], impairing the photosynthesis of plants. Plants have adapted to high-temperature climates by evolving several heat-resistant mechanisms. Under heat stress, the levels of corresponding metabolites and proteins involved in the expressions of genes related to membrane stability, heat shock proteins, heat shock transcription factor, and upregulated antioxidants were increased accordingly [6,7,8,9]. In plants, high-temperature signals are transmitted into cells through transduction pathways, accumulating unfolded proteins and ROS, and thus regulating the expression of transcription factors (TFs) and key heat-resistant genes [10,11,12]. Heat shock transcription factors (HSFs) are the main regulators used by plants in response to heat stress. HSFs are distributed along the heat shock response signaling pathway and are involved in the transcriptional regulatory network in response to heat stress. HSF binds to the heat shock element (HSE) to trigger the heat shock protein (HSP), improving plant thermotolerance [13,14]. Classical plant hormones can integrate environmental stimuli and endogenous signals to regulate plant defense in responses to various abiotic stresses, including heat stress. Exogenous applications of these hormones can likely improve the plant’s thermotolerance [15]. At present, transcriptome sequencing has been widely applied in order to study various biological processes, explore gene expression changes in response to abiotic stress, and to identify transcription factors [16]. De novo transcriptome sequencing of watermelon revealed that genes involved in nitrogen metabolism, phenylpropane metabolism, and photosynthesis play important role underlying NO-induced aluminum stress alleviation in watermelons [17]. Transcriptome analysis of two blueberry species under heat stress revealed several important biosynthesis and metabolic pathways which are crucial in response to heat stress [18].

Melatonin (N-acetyl-5-methoxytryptamine, MT) is an essential indoleamine for animals’ and plants’ vital activity and biological processes [19,20]. In plants, MT can promote seed germination [21], fruit development, and ripening regulation [22,23], thus improving fruit quality and yield [24]. MT is also a highly efficient endogenous free radical scavenger with antioxidant properties [25]. Moreover, exogenous MT improves the activities of superoxide dismutase (SOD), peroxidase (POD), catalase (CAT), and ascorbate peroxidase (APX), strengthening the plant’s stress-tolerance ability [26]. On the other hand, exogenous MT regulates the transcription levels of the key genes (bZIP, bHLH, WRKY, MYB, HSPs, etc.) involved in signal transduction and metabolic pathways in plants under abiotic stress. It can protect plants from extreme temperatures [27,28], drought [29], salinity [30], and heavy metal exposure [31]. A study on strawberry plants showed that spraying exogenous MT can upregulate the expression of the HSF genes and protect strawberries from heat damage by inducing the *HSFA2a*, *HSFB1a*, and *HSP90* gene expressions [32]. In *Arabidopsis*, MT triggered the heat-response genes *HSA32*, *HSFA2*, *HSP90*, and *HSP101*, strengthening heat tolerance. Therefore, MT-induced regulatory genes are likely an important part of the molecular mechanism that produces heat tolerance in plants [28]. 

Celery (*Apium graveolens* L.) is a biennial herb of the Apiaceae family from the Mediterranean and Middle East regions, and it is cultivated worldwide [33]. It is rich in vitamins, carotene, protein, and cellulose. Celery is also a good source of flavonoids, volatile oils, and antioxidants, and it contains various bioactive ingredients, notably apigenin and terpenoids. Celery grows in a low-temperature climate (15–20 °C). Climate temperature above 35 °C can cause protein degeneration and membrane damage, impairing physiological and biochemical metabolism. The high temperature leads to the yellowing, browning, wilting, or burning of leaves, inhibited root growth, and ultimately reduces the economic value of the celery [34]. Therefore, measures to reduce high-temperature damage to celery are needed. Exogenous MT can reduce plant damage caused by high temperatures, but MT usage in celery has not been studied. In this study, ‘Liuhe Huangxinqin’ was used as the plant model to investigate the mechanism of exogenous MT on celery seedlings under heat stress. The plants’ physiological, anatomical, transcriptional, and molecular-level changes were observed in order to understand the mechanism of MT-induced heat stress resistance in celery. This study has preliminarily revealed the effect of MT on the gene expression regulation of celery seedlings under heat stress, and has provided a theoretical basis for the rational use of MT to alleviate thermal damage during celery growth. 

## 2. Results

### 2.1. Growth and Physiological Analysis under Heat Stress

**Figure 1:**
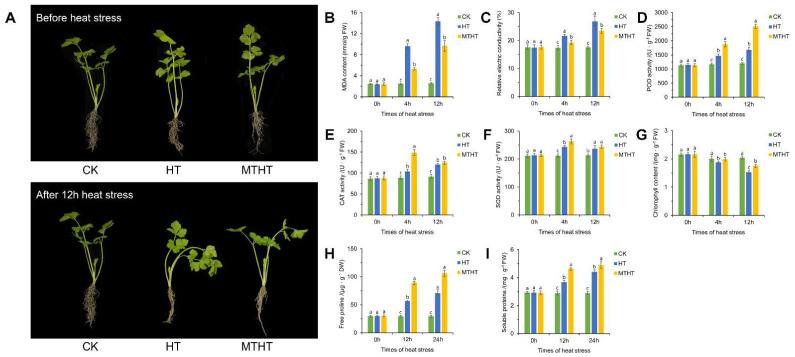
Effects of heat stress on celery. (**A**) Phenotypic characteristics of celery seedlings under heat stress. (**B**–**I**) Effects of heat stress on physiological characteristics of celery. (**B**) Malondialdehyde (MDA), (**C**) relative conductivity, (**D**) peroxidase (POD), (**E**) catalase (CAT), (**F**) superoxide dismutase (SOD), (**G**) chlorophyll content, (**H**) free proline content, and (**I**) soluble protein. Each error line represents the mean ± SD, *t*-test with significance level of 0.05 (*p* < 0.05). The different letters (a–c) indicate a significant difference, and the same letter indicates no significant difference. CK: non-treatment group, HT: water-sprayed group, MTHT: 100 μmol·L^−1^ MT-sprayed group.

As is shown in Figure 1A, celery seedlings under different treatments exhibited different heat stress responses after 12 h heat stress. Seedlings of CK (non-treatment group) and HT (water-sprayed group) were severely dehydrated at both the petiole and the leaves, the petiole was bent, there was leaf shrinkage, and there was slight yellowing at the leaf edges. Seedlings treated with exogenous MT solution exhibited significantly reduce heat damage compared with the other treatments. The MDA and REC of MTHT (100 μmol·L^−1^ MT-sprayed group) seedlings showed lower physiological indicators, measured at three stages under heat stress, than the HT seedlings under heat treatment (Figure 1B, C). Concurrently, the activities of antioxidant enzymes, such as POD, CAT, and SOD, were significantly increased (Figure 1D–F). The chlorophyll, free proline, and soluble protein content in MTHT seedlings were notably higher than in HT seedlings (Figure 1G–I). Exogenous MT treatment successfully maintained celery morphology and improved photosynthetic pigment stability in the celery seedlings. The results indicate that cytoplasmic membrane damage under heat stress can be relieved by enhancing the osmotic adjustment ability and ROS-scavenging ability of celery seedlings, and by reducing the extravasation of electrolytes, effectively reducing the damage of high temperature to celery seedlings. 

### 2.2. Changes in Stomatal Movement and Cross-Section of Celery Leaves under Heat Stress

**Figure 2:**
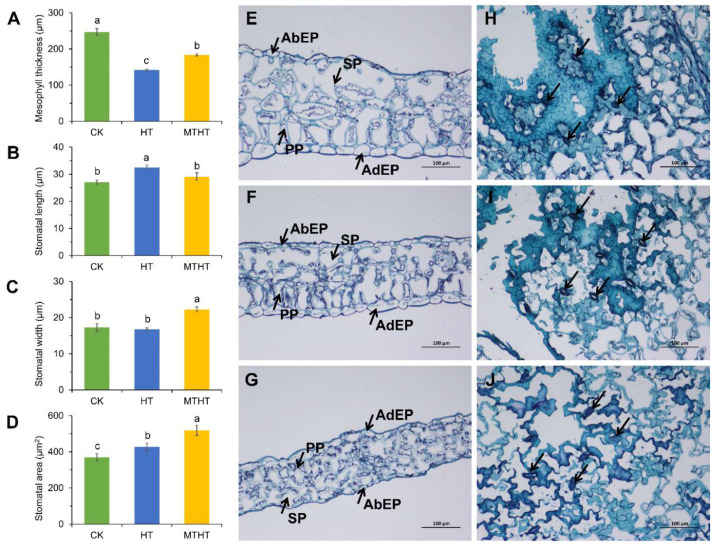
Anatomical characteristics of celery leaves under heat stress. (**A**) Mesophyll thickness, (**B**) stomatal length, (**C**) stomatal width, (**D**) stomatal area, (**E**–**G**) leaf section, and (**H**–**J**) leaf plan. SP: spongy tissue; PP: fence organization; AdEp: adaxial epidermal cell layer; AbEp: abaxial epidermal cell layer. Arrows in figure (**H**–**J**) point to stomata. Each error line represents the mean ± SD, and *t*-test with significance level of 0.05 (*p* < 0.05). Different letters (a–c) indicate significant differences, while the same letter indicates no significant differences.

The morphology and stomatal movement of celery leaves under heat stress were observed. After 4 h of heat stress, the mesophyll thickness of the leaves treated with MTHT and HT was significantly reduced compared with those of CK (Figure 2A,E–G). The spongy and palisade tissue in CK and MTHT leaves were arranged in regular shapes, and the upper and lower epidermal cells were full-shaped. In HT leaves, spongy, palisade, and upper and lower epidermal cells became thin with severe cell dehydration (Figure 2E–G). The stomata also varied significantly among the three treatment groups (Figure 2B–D, H–J). In response to heat stress, leaves treated with MTHT increased their transpiration rate with the stomatal aperture opened. As a result, the width, length, and area of the stomata were higher than those of CK. In contrast, HT leaves were dehydrated and wilted, having lost water in guard cells, resulting in the stomata closing. The width and area of HT stomata were lower than those of CK.

### 2.3. Transcriptome Sequencing and Assembly

To further investigate the mechanism of reducing MT-induced heat stress, nine cDNA libraries were constructed for transcriptome sequencing from three groups of samples, including three biological replicates. A total of 61.71 G of raw data were obtained. The average Q20 and Q30 were greater than 96.92% and 91.67%, respectively. The average similarity rate between each sample and the reference was 91.11% (Table S1). 

To detect the reproducibility of transcriptome sequencing, a biological replicate correlation analysis of the FPKM values of each sample was performed (Figure S1). The average correlation coefficients of the FPKM values between the three treatment replicates were 0.954, 0.968, and 0.944, indicating that the reproducibility of the samples in the group was better. The correlation of three biological replicates in the two different sets of heat treatments was higher than in the others, suggesting that there were differentially expressed genes in different heat treatments. Principal component analysis (PCA) showed that there was some degree of aggregation between the biological replicates of the three treatments. However, there was a significant separation of the biological replicates of different treatments, indicating that there were large differences between the groups. 

### 2.4. Differentially Expressed Genes (DEGs) Analysis

**Figure 3:**
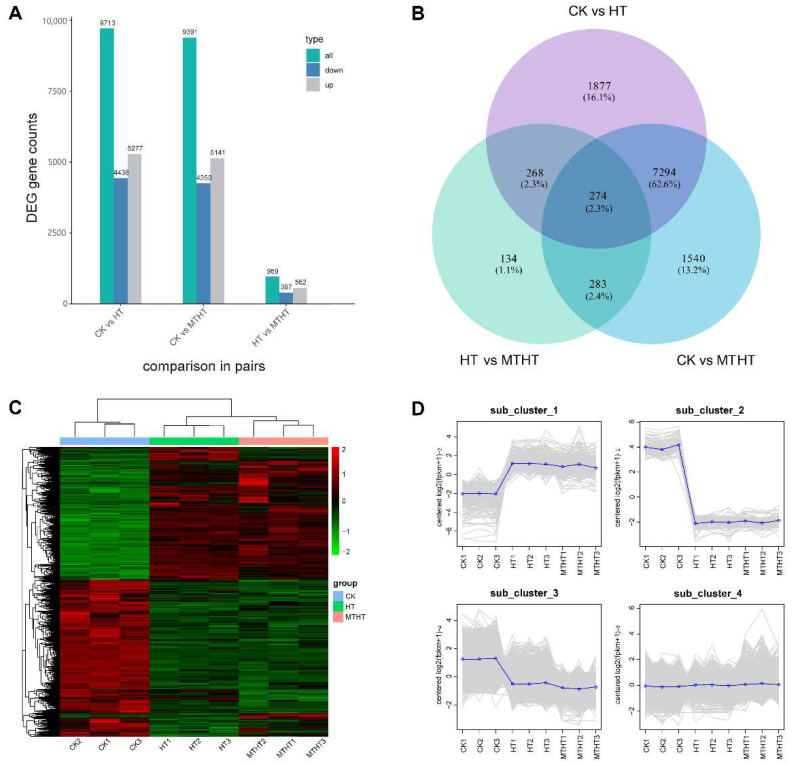
Distribution characteristics of differentially expressed genes (DEGs) in three different comparison groups. (**A**) The number of DEGs for any two different comparisons. Green indicates the number of all the differential genes, blue indicates the downregulated differential genes, and gray indicates the upregulated differential genes. (**B**) The Venn plot represents the number and proportion of DEGs that are commonly or uniquely expressed in pairwise comparisons. (**C**) Clustering of the DEGs’ transcript abundance in all samples. (**D**) K-means clustering of gene expression trends. The expression profile of each gene in each subpopulation is shown as a gray line, and the average expression profile of all the genes in each sample is shown in blue.

Among the screened DEGs, there were 9713 DEGs in the CK vs. HT comparison pair, 5277 of which were shown to be upregulated, and 4436 of which were downregulated. Among CK vs. MTHT, 9391 DEGs were commonly expressed, 5141 of which were upregulated, and 4250 of which were downregulated. About 959 DEGs in HT vs. MTHT were identified, with 562 upregulated DEGs and 397 downregulated DEGs (Figure 3A). Two or more comparisons analyzed three comparison pairs for common and uniquely expressed genes. Figure 3B shows the largest number of commonly detected DEGs between the CK vs. HT and CK vs. MTHT comparison pairs, a total of 7568, which is 64.8% of the total. The number of DEGs in CK vs. MTHT was 337 fewer than in CK vs. HT due to the administration of exogenous MT. There were only 134 DEGs present in HT vs. MTHT, indicating the key genes in response to heat stress under MT induction.

Figure 3C shows the hierarchical clustering heatmap created based on each sample’s expression matrix, which illustrates the expression patterns of the DEGs across the three treatments. All DEGs were divided into two groups with three replicates. The genes of group 1 were highly expressed in HT and MTHT, while genes of group 2 were highly expressed in CK. However, several gene expression levels varied significantly among HT and MTHT. K-means cluster analysis was performed to further classify the expression patterns of all DEGs in each sample, as shown in Figure 3D. Four subclasses were identified, clusters 1 to 4, with different average expressions. Cluster 1 contained 506 genes highly expressed in HT and MTHT, with expression levels in MTHT lower than in HT. Gene expression levels in cluster 2 and cluster 3 were decreased. The expression levels of 81 genes among cluster 2 and 2512 genes among cluster 3 decreased gradually. Cluster 4 contained 8571 genes with stable expression levels and with a slight increase. MT could likely regulate some of the DEGs, alter their expression patterns, and determine the celery heat-tolerance response.

### 2.5. Analysis of Differentially Expressed Transcription Factors (TFs)

**Figure 4:**
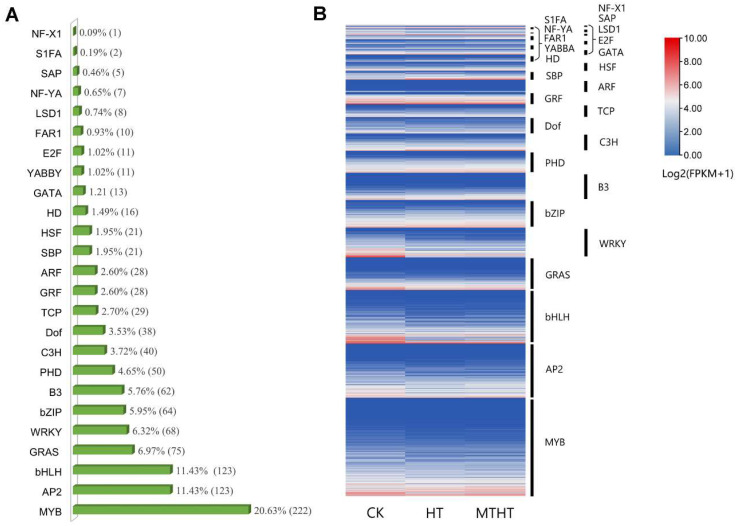
Celery transcription factor under heat stress. (**A**) Differentially expressed transcription factor genes. The percentage is the ratio of the number of members of each transcription factor family to the total number of members of all celery transcription factors. (**B**) Specific expression of transcription factors. Colors from blue to red represent expression levels from low to high, which were calculated using log2 (FPKM+1). The heat maps label the family of transcription factors.

The TFs of the DEGs were analyzed and named using transcription factor family information from the Plant Transcription Factor database. A total of 368 differentially expressed TFs were divided into 23 families, including MYB, AP2/ERF, bHLH, GRAS, WRKY, etc. (Figure 4A). The families with the largest number of transcription factor-containing genes were MYB (*n* = 222), AP2 (*n* = 123), and bHLH (*n* = 123). As the heatmap from the gene expression levels of each TF family member shows, the TFs of MYB, bHLH, and GRF were highly expressed in all treatments. The SBP and GATA transcription families were significantly expressed only in HT vs. MTHT, which was likely induced by MT, as shown in Figure 4B.

### 2.6. DEGs Annotation Analysis

**Figure 5:**
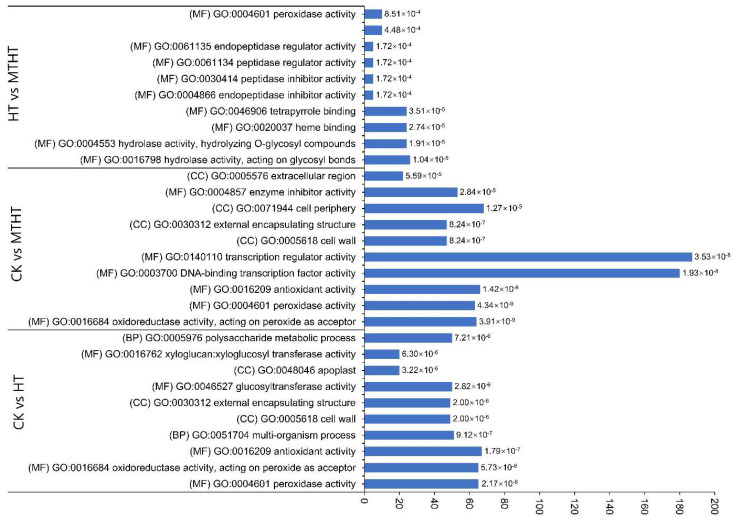
Gene Ontology (GO) enrichment analysis of DEGs among comparison pairs. The top 10 enriched terms with highly significant *p*-values (≤0.05) in each comparison are represented. The values of the false discovery rate are shown in the bar graph. (BP): biological process; (MF): molecular function; (CC): cellular component.

Gene ontology (GO) analysis was used to classify DEGs annotations among the three comparison pairs to further analyze DEGs’ biological functions. The first ten significantly enriched annotation terms were selected, including the three main GO categories of cell components (CCs), biological processes (BPs), and molecular functions (MFs), as shown in Figure 5. Most DEGs were enriched in molecular functions, followed by cellular components, in which the transcription regulator activity (GO:0140110) contains the largest number of genes. Of the cell components, “cell wall” and “external encapsulation structure” were found in CK vs. HT and CK vs. MTHT. Exogenous MT likely caused changes in cell structure to relieve the cell damage from the heat. Notably, peroxidase activity (GO: 0004601), endopeptidase regulator activity (GO: 0061135), and peptidase regulatory activity (GO: 0061134) exhibited distinctive enrichment patterns in HT vs. MTHT. Exogenous MT is likely involved in oxidative reactions by regulating the activity of antioxidant enzymes, peptidases and endopeptidases to reduce oxidative damage to cells

**Figure 6:**
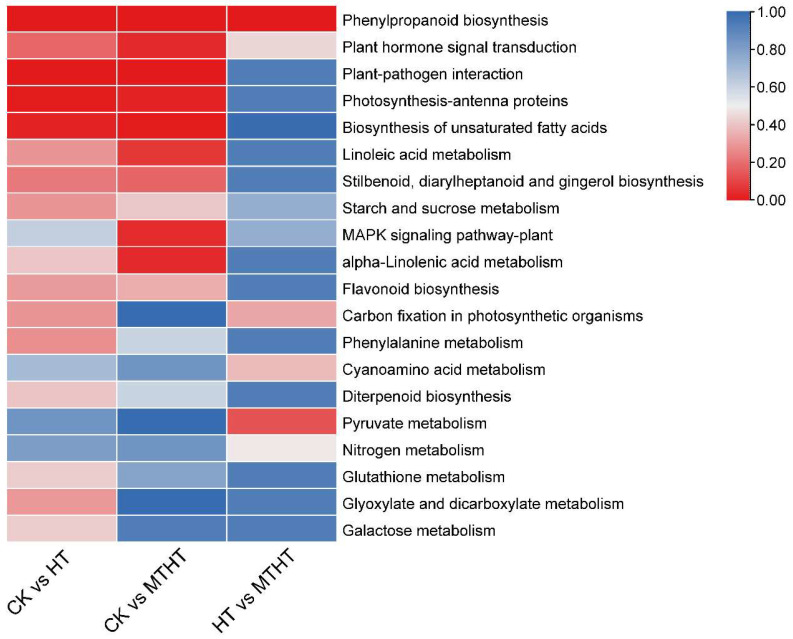
Kyoto Encyclopedia of Genes and Genomes (KEGG) enrichment analysis of DEGs among comparison pairs. The plot is drawn based on the q value of the enrichment in the pathway, and a redder color indicates that the enrichment is more pronounced. The first 20 enrichment pathways are represented. Significant DEGs are identified based on a false discovery rate (FDR) ≤ 0.05 and a |log2 ratio| ≥ 1).

The DEGs were further mapped to the KEGG database in order to investigate the metabolic pathways. Twenty metabolic pathways with significant expression were selected to make a heat map, as shown in Figure 6. KEGG annotations were largely found in the “Phenylpropanoid biosynthesis” and “Plant hormone signal transduction” pathways among the three comparison pairs. Nevertheless, “Plant-pathogen interaction”, “Photosynthesis-antenna proteins”, “Biosynthesis of unsaturated fatty acids”, “Linoleic acid metabolism”, “Stilbenoid”, “diarylheptanoid and gingerol biosynthesis”, and “Starch and sucrose metabolism” were abundant in CK vs. HT and CK vs. MTHT. Notably, the “Pyruvate metabolism”, “Cyanuric acid metabolism”, and “Nitrogen metabolism” pathways were only found in significant amounts in the HT vs. MTHT comparison pair. Thus, applying MT decreases the damage of heat stress on celery seedlings by altering the pyruvate metabolism, the cyanuric acid metabolism, and the nitrogen metabolism. 

### 2.7. Special Metabolic Pathway of Celery in Response to Heat Stress

**Figure 7:**
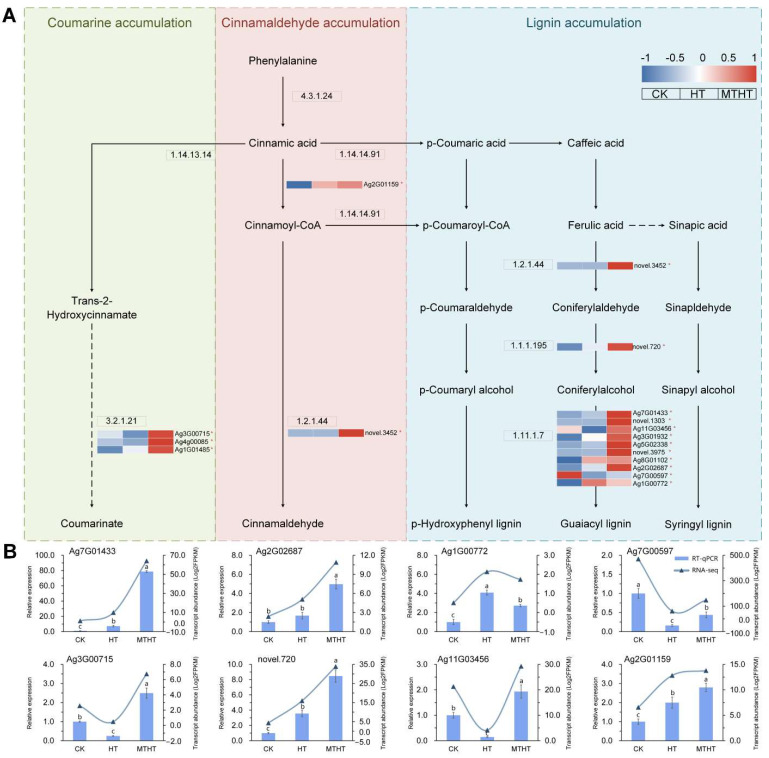
(**A**) Metabolite transformation and gene expression changes in the pyruvate metabolism pathway under heat stress. Red (up-tuned) and blue (down-tuned) in the heat map represent gene expression trends. * mean the gene is DEGs. (**B**) RT-qPCR analysis of genes in key pathway under heat treatment. Different letters (a–c) indicate significant differences, while the same letter indicates no significant differences. The column shape represents the relative expression of genes in RT-qPCR, and the curve with triangles represents the RNA-sequencing data.

Two heat-stress responsive metabolic pathways of celery treated with MT were identified through function annotation (Table S2). In the pyruvate metabolic pathway, as displayed in Figure 7A, enzyme-coding genes in the synthesis pathway from glucose to pyruvate were upregulated in MTHT by exogenous MT, including pyruvate kinase (2.7.1.40), phosphofructokinase-1 (2.7.1.90), and phosphoglycerate kinase (2.7.2.3). Most of the enzyme-coding gene expression in the pyruvate metabolic pathway participating in the TCA cycle showed a reducing trend. The expression of two key enzyme-coding genes in pyruvate consumption, pyruvate dehydrogenase (4.1.1.31) and isocitrate dehydrogenase (NADP^+^) (1.1.1.42), was significantly downregulated in the TCA cycle, as well as other genes associated with pyruvate consumption. The induction of exogenous MT altered the pyruvate metabolizing enzymes and caused the accumulation of pyruvate in cells, which is involved in ROS-scavenging. Eight genes in the pathway were selected for RT-qPCR validation, as shown in Figure 7B. The three key enzymes in the pyruvate synthesis pathway were phosphoglycerate kinase and pyruvate phosphofructokinase-1 genes (*Ag3G00009*, *Ag2G02897*, *Ag2G02950*). Their expression levels in MTHT were significantly increased by 4.65, 2.72, and 1.53 times compared with HT. Pyruvate dehydrogenase (*Ag3G01239*), which plays a role in the mitochondrial synthesis of acetyl-CoA, decreased significantly in MTHT after MT application. In the TCA cycle, the expression of aconitate hydratase (*Ag9G00972*), isocitrate dehydrogenase (NADP^+^) (*Ag10G02317*), and malate dehydrogenase (*Ag4G00362*) decreased in both HT and MTHT. These results are in agreement with the gene expression patterns in transcriptome sequencing.

**Figure 8:**
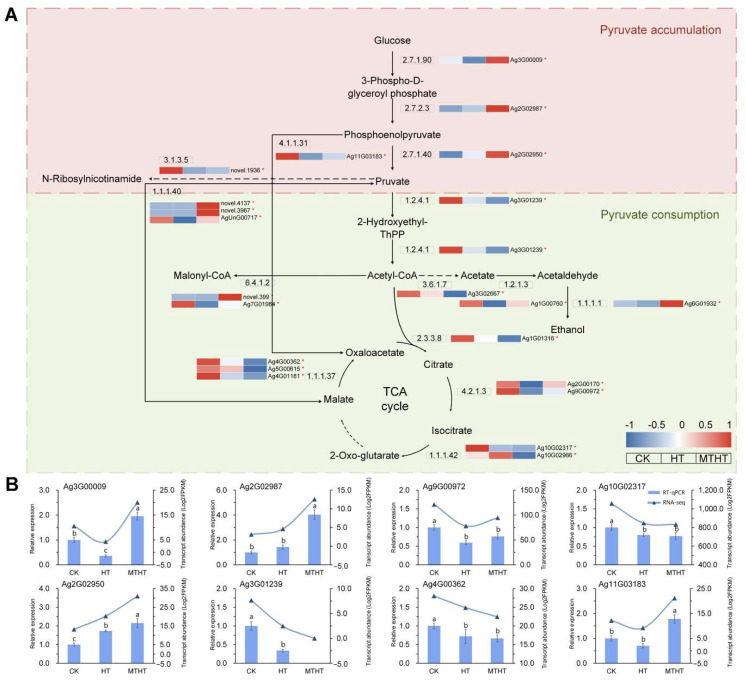
(**A**) Metabolite transformation and gene expression changes in the phenylpropanoid biosynthesis pathway under heat stress. Red (up-tuned) and blue (down-tuned) in the heat map represent gene expression trends. * mean the gene is DEGs. (**B**) RT-qPCR analysis of genes in key pathway under heat treatment. Different letters (a–c) indicate significant differences, while the same letter indicates no significant differences. The column shape represents the relative expression of genes in RT-qPCR, and the curve with triangles represents the RNA-sequencing data.

Peroxidase was detected in the phenylpropanoid biosynthesis pathway (Figure 8A) and linked to the synthesizing of three antioxidants: coumarin, cinnamaldehyde, and lignin. After exogenous MT application, the expression of all coding genes increased in the pathways of β-glucosidase (3.2.1.21), trans-cinnamate 4-monooxygenase (1.14.14.91), cinnamyl-coenzyme A reductase (1.2.1.44), and cinnamyl alcohol dehydrogenase (1.1.1.195), improving the efficiency with which the phenyl propionic acid metabolism synthesized antioxidants. The expression of nine coding genes in peroxidase (1.11.1.7) was upregulated significantly by MT, where expression of *Ag7G01433* was upregulated about seven-times. Applying exogenous MT could improve the peroxidase activity of celery seedlings, improving their ability to remove ROS, and reducing heat stress. Eight genes were chosen for RT-qPCR in the phenylpropanoid biosynthesis pathway, as shown in Figure 8B. In the MT-induced MTHT, the expression of the peroxidase encoding genes *Ag7G01433*, *Ag2G02687*, *Ag7G00597*, and *Ag11G03456* were upregulated, increasing the peroxidase activity. The expression of three other genes in the metabolic pathway, *Ag3G00715*, *novel.720*, and *Ag2G01159*, was also upregulated after MT application, promoting the synthesis of antioxidants. These results agree with the physiological indicators and transcriptomics sequencing results of previous experiments.

**Figure 9:**
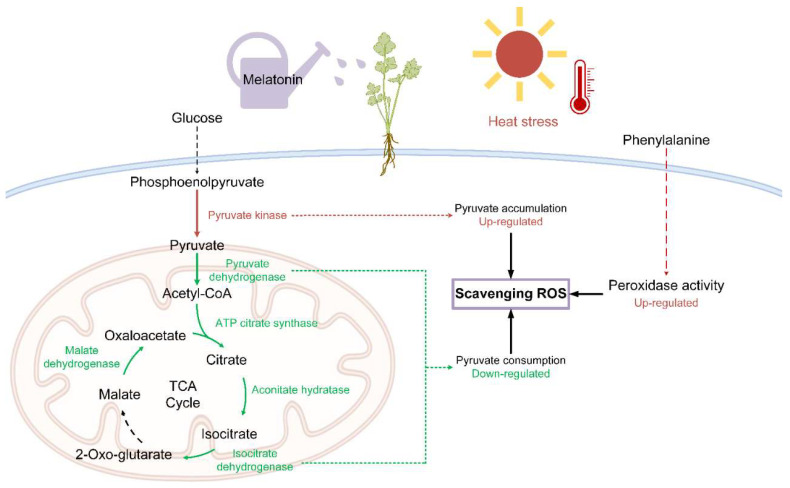
Schematic diagram of two pathways for scavenging reactive oxygen species (ROS) in celery under heat stress. In red, expression is elevated, and in green, expression is reduced.

Hence, exogenous MT enhanced the antioxidant enzyme activity in celery seedling cells, promoted the accumulation of pyruvate, enhanced the synthesis of antioxidants, rapidly removed ROS, and relieved the oxidative stress of cells, protecting the celery seedlings from heat stress, as shown in Figure 9.

## 3. Discussion

With the intensification of global warming trends, heat stress has become an important abiotic stress faced by plant cultivators [35]. Understanding the mechanisms by which plants resist heat stress is crucial for mitigating the problem. Exogenous MT treatment can strengthen plant tolerance to abiotic stresses [36], including heat stresses when exposed to high temperatures. The plants respond to heat stress by altering their physiological and transcriptional aspects. In the two heat-stress treated groups (HT and MTHT), the antioxidant enzyme activity, chlorophyll content, proline content, and protein content of the plant cells were improved. However, MT pretreatment significantly reduced the MDA content and relative conductivity in the MTHT samples. In a study on soybeans (*Glycine max*) under low-temperature stress, exogenous MT was observed to strengthen cold-tolerance by increasing mineral elements and osmotic regulators and reducing MDA accumulation [26]. Exogenous MT can increase leaf length and width, as well as the number of stomata [37]. The MTHT group showed wider stomata apertures, higher leaf thickness, palisade tissue, and sponge tissue, keeping the temperature and water loss stable and thus relieving heat stress.

Similarly, heat tolerance studies on *Rhododendron simsii* [38] and *Lolium perenne* L. [39] have shown that varied leaf thickness and large stomatal density can effectively maintain low stomatal obstruction of leaves at high temperatures for a long time, strengthening their thermotolerance. Other studies have found that, in *Citrus maxima* [2], *Brassica oleracea* [40], and *Lycopersicon esculentum* [41], heat stress can cause damage to subcellular structures such as chloroplast membranes. Alterations in chloroplast ultrastructure directly causes the deterioration of the photosynthetic rate of plants. Exogenous MT can protect the chloroplast matrix layer and leaf structure [42], maintaining cell expansion and water-holding capacity, preventing photosynthetic pigment degradation [43], and promoting PS II electron transport. The chlorophyll content of the MTHT celery seedlings was higher than that of the HT seedlings, enhancing photosynthesis and light energy utilization efficiency.

GO analysis showed that, in molecular function, the DEGs are mainly enriched in “transcription regulator activity”, which is responsible for regulating gene expression in the transcription step and controlling the gene transcription activity. The “cell wall” and “external encapsulation structure” were enriched in the cell composition, indicating that under heat stress the content and structure of the cell wall components and the mechanical properties of the cell wall were altered. In studies of *Diplocarpon mali* in apples [44] and *Peronophythora litchi* in lichees [45], administrating MT can accelerate lignin accumulation and the lignification of cell walls by increasing polyphenol oxidase (PPO) enzyme activity, reducing the damage caused to plants by pathogenic bacteria infestation. In MTHT, the rising endopeptidase and peptidase activities accelerated the rate of protein decomposition into amino acids. They reduced the accumulation of oxidized proteins and H_2_O_2_, strengthening the resistance of the plants to heat stress. There were 23 transcription families identified among the DEGs, including the heat stress-related families HSF, WRKY, MYB, AP2, bZIP, and bHLH. The expression of the BSP and GATA family members in MTHT was abundant compared with HT. Studies have shown that in *Camellia japonica* L. [46] and *Capsicum annuum* L. [47], BSP family genes can aid in mitigating abiotic stress via signaling pathways of hormones such as ABA, GA, MeJA, etc., strengthening the stress tolerance of plants. The GATA family can also respond to heat stress. In rice, the *TaGATA54*, *TaGATA57*, and *TaGATA60* genes exhibit high expression levels when exposed to high temperatures [48].

The “Pyruvate metabolism” and “Phenylpropanoid biosynthesis” pathways were significantly enriched in MTHT, as indicated via KEGG analysis. Under heat stress, O^2−^ and H_2_O_2_ are produced in mitochondria and chloroplasts, and the ROS level in the cytoplasm and nucleus increases [49]. ROS aid in the perception of high-temperature stress and the triggering of the plant’s defense mechanisms. POD enzymes help produce and detoxify hydrogen peroxide by oxidizing phenolic compounds to maintain intracellular ROS levels. POD enzymes are an important antioxidant and initial defense against abiotic stress [4]. Exogenous MT increases the activity of antioxidant enzymes (SOD, POD, CAT, and APX) and non-enzymatic antioxidant substances (AsA and GSH). It also upregulates the SOD, POD, CAT, APX, and other gene expressions by inhibiting ROS production [50,51,52], and reducing both lipid peroxidation and the oxidative stress caused by impairments to plant growth and development. MT has shown to upregulate the *catalase* (*SlCAT1*), *ascorbate peroxidase* (*SlcAPX*), and *glutathione reductase 1* (*SlGR1*) expressions in tomatoes [50]. AtGs (*ATG2*, *ATG5*, and *ATG18a*), *NHX1*, and *SOS1* in *Arabidopsis* were also affected by exogenous MT, strengthening plant salinity tolerance [52]. Previous studies have proved that heat stress inhibits pyruvate content, removing the ROS produced by thermal stress in a non-enzymatic form, and protecting cells from damage [53]. This study has found that altering the expression level of pyruvate metabolism genes influences the accumulation of pyruvate. Undoubtedly, heat stress alters various biological pathways, and this needs further investigation.

## 4. Materials and Methods

### 4.1. Plant Material and Treatment

In this study, a heat-sensitive celery cultivar, ‘Liuhe Huangxinqin’, was used as plant material. The seeds were placed in a moist petri dish and kept in an incubator at 20 °C in the dark for a germination environment. After most of the seeds had germinated, the seeds were transferred into the seedling tray with a temperature of 25/20 °C (day/night) and photoperiod of 12 h/12 h (day/night). When the celery seedlings grew 2~3 true leaves, the robust seedlings were transplanted to a 10 cm diameter nutrient bowl for routine management in the open air. The 100 μmol·L^−1^ MT solution was prepared as follows: 23.7 mg of MT (Sigma-Aldrich New Zealand Co, Darmstadt, Germany) was dissolve in 1.0 mL of absolute ethanol, and then the solution was transferred to a 1 L volumetric flask and distilled water was added to volume. The treatment started when the celery seedlings grew to a plant height of about 15 cm, and the celery was divided into three groups: CK (non-treatment group), HT (water-sprayed group), MTHT (100 μmol·L^−^^1^ MT-sprayed group). Both the water and MT solution were continuously sprayed for 3 d on the treatment groups. Plant materials were moved to a culture incubator with a temperature of 38 °C and humidity of 80%, and celery leaf samples were collected after 0, 4, and 12 h of treatment, with nine biological replicates for each treatment. The samples were stored in the refrigerator at −80 °C after quick freezing with liquid nitrogen. 

### 4.2. Physiological Indicators Assay and Leaf Structure Observation

MDA content was measured using thiobarbituric acid (TBA), and the relative conductivity (REC) was measured using a conductance meter. The SOD, CAT, and POD activities were quantified using nitroblue tetrazolium (NBT), the potassium permanganate titration method, and the guaiacol method, respectively. The celery leaves were added to a 50% FAA fixating solution (formalin: glacial acetic acid: 50% ethanol = 1:1:18), fixed at 4 °C for 24 h, dehydrated with ethanol, embedded in paraffin, and stained with safranin O/fast green (1%/0.5%). The leafy cross-sectional features of the blades were observed under a light microscope, and images were collected and analyzed using an imaging system (Nikon DS-U3, Nikon, Tokyo, Japan), and ImageJ software was used (version 1.8.0.112; National Institutes of Health: Bethesda, MD, USA, 1997) to analyze the stomatal features.

### 4.3. RNA Extraction, Library Construction, Sequencing, and Data Assembly

Plant RNA was extracted using the Trizol method, followed by strict quality control of RNA samples using Agilent 2100 bioanalyzer to accurately detect RNA integrity and concentration. The mRNA with polyA tails were enriched with Oligo (dT) magnetic beads, and then randomly interrupted mRNA with divalent cations in a fragmentation buffer. Using the fragmented mRNA as a template and random oligonucleotide as a primer, the first strand of cDNA was synthesized in the M-MuLV reverse transcriptase system, and then the RNA was degraded with RNaseH. Under the DNA polymerase I system, the second strand of cDNA was synthesized using dNTPs as raw materials. The purified double-stranded cDNA was end repaired, polyA tails were added, and it was ligated with adaptors. To create the final cDNA library, fragments of 250–350 bp in length were selected using the AMPure XP system (Beckman Coulter, Beverly, CA, USA) for PCR amplification. The kit NEBNext^®^ Ultra™ RNA Library Prep Kit for Illumina^®^ was used for library construction. Subsequently, the insert size of the library was detected using Agilent 2100 bioanalyzer. Illumina sequencing was performed after pooling different libraries according to the effective concentration and the demand of the target data volume. Four fluorescently labeled dNTP, DNA polymerase, and linker primers were added to the sequencing flow cell for amplification, and the sequencer obtained the sequence information of the fragment to be measured by capturing the fluorescence signal and converting the optical signal into a sequencing peak. Transcriptome sequencing was completed by Nuohe Gene Technology Co., Ltd., Beijing, China. 

### 4.4. Screening and Classification of Differentially Expressed Genes (DEGs)

The transcript abundance value (fragments per kilobase of transcripts per million mapped fragments, FPKM) of each gene was calculated using FeatureCounts (1.5.0-p3). After the quantification of gene expression was completed, the expression data was statistically analyzed, and genes with significantly different expression levels in variable states were screened. Analysis of differentially expressed genes between two comparative combinations was performed using the DESeq2 software (version 1.20.0, Bioconductor, Boston, MA, USA). Benjamini and Hochberg’s FDR-controlling procedure [54] was used to adjust the resulting *p*-value to control the error discovery rate. Differential analyses were performed using DESeq2, and the DEGs were filtered using padj ≤ 0.05 and |log2FlodChange| ≥ 1 as a threshold.

### 4.5. Validation of Gene Expression Levels

Celery RNA was extracted using the plant total RNA extraction kit Plant Total RNA Isolation Kit (Goldenstar RT6 cDNA Synthesis Kit Ver.2 Ltd.). cDNA was synthesized using a Goldenstar RT6 cDNA Synthesis Kit Ver. 2 (Beijing TsingKe Biotech Co., Ltd., Beijing, China). RT-qPCR was performed using the real-time PCR system Bio-Rad CFX96TM (Bio-Rad, Hercules, CA, USA) with the 2× T5 Fast qPCR Mix (SYBR GreenI) (TsingKe, Beijing, China). Gene-specific primers were designed using Primer Premier 6 and are listed in Table S3. 

## 5. Conclusions

In this study, exogenous MT treatment was shown to protect celery seedlings against heat stress. Exogenous MT may protect celery seedlings’ photosynthetic mechanism, preserve their photosynthetic pigments, and enhance their photosynthetic performance. In addition, MT effectively reduced heat-induced oxidative stress by improving the antioxidant enzyme activity and antioxidant system, increasing pyruvate accumulation and reducing ROS accumulation, inducing stress-resistant gene expression, and strengthening the ability of the celery to tolerate high temperatures. Overall, the physiological and molecular mechanisms of MT regulating celery in response to heat stress provide a new perspective for cultivating celery under stress, and for cultivating high-quality heat-resistant germplasm.

## Data Availability

Transcriptional and metabolic data were generated by Nuohe Gene Technology Co., Ltd., and physiological and anatomic metabolic data were generated by the authors themselves.

## Supplementary Materials

The following supporting information can be downloaded at: https://www.mdpi.com/article/10.3390/ijms231911382/s1.

## References

[1] Schiermeier Q. (2018). Droughts, heatwaves and floods: How to tell when climate change is to blame. Nature.

[2] Chen W.R., Zheng J.S., Li Y.Q., Guo W.D. (2012). Effects of high temperature on photosynthesis, chlorophyll fluorescence, chloroplast ultrastructure, and antioxidant activities in fingered citron. Russ. J. Plant Physiol..

[3] He Y., Huang B.R. (2010). Differential responses to heat stress in activities and isozymes of four antioxidant enzymes for two cultivars of kentucky bluegrass contrasting in heat tolerance. J. Am. Soc. Hortic. Sci..

[4] Kidwai M., Ahmad I.Z., Chakrabarty D. (2020). Class III peroxidase: An indispensable enzyme for biotic/abiotic stress tolerance and a potent candidate for crop improvement. Plant Cell Rep..

[5] Haque M.S., Kjaer K.H., Rosenqvist E., Sharma D.K., Ottosen C.O. (2014). Heat stress and recovery of photosystem II efficiency in wheat (*Triticum aestivum* L.) cultivars acclimated to different growth temperatures. Environ. Exp. Bot..

[6] Wang X.M. (2005). Regulatory functions of phospholipase D and phosphatidic acid in plant growth, development, and stress responses. Plant Physiol..

[7] Zhang Q., Geng J., Du Y.L., Zhao Q., Zhang W.J., Fang Q.X., Yin Z.G., Li J.H., Yuan X.K., Fan Y.R. (2022). Heat shock transcription factor (Hsf) gene family in common bean (*Phaseolus vulgaris*): Genome-wide identification, phylogeny, evolutionary expansion and expression analyses at the sprout stage under abiotic stress. BMC Plant Biol..

[8] Tian F.X., Hu X.L., Yao T., Yang X.H., Chen J.G., Lu M.Z., Zhang J. (2021). Recent advances in the roles of HSFs and HSPs in heat stress response in woody plants. Front. Plant Sci..

[9] Zhang J., Chen H.Y., Wang H.H., Li B., Yi Y.J., Kong F.J., Liu J.Y., Zhang H.X. (2016). Constitutive expression of a tomato small heat shock protein gene *LeHSP21* improves tolerance to high-temperature stress by enhancing antioxidation capacity in Tobacco. Plant Mol. Biol. Rep..

[10] Mittler R., Finka A., Goloubinoff P. (2012). How do plants feel the heat?. Trends Biochem. Sci..

[11] Bokszczanin K.L., Fragkostefanakis S., (SPOT-ITN) Consortium (2013). Perspectives on deciphering mechanisms underlying plant heat stress response and thermotolerance. Front. Plant Sci..

[12] Yan Q.J., Huang Q., Chen J.B., Li J.X., Liu Z.B., Yang Y., Li X.F., Wang J.M. (2017). *SYTA* has positive effects on the heat resistance of *Arabidopsis*. Plant Growth Regul..

[13] Anckar J., Sistonen L. (2011). Regulation of HSF1 function in the heat stress response: Implications in aging and disease. Annu. Rev. Biochem..

[14] Zahid K.R., Ali F., Shah F., Younas M., Shah T., Shahwar D., Hassan W., Ahmad Z., Qi C., Lu Y.L. (2016). Response and tolerance mechanism of cotton *Gossypium hirsutum* L. to elevated temperature stress: A review. Front. Plant Sci..

[15] Li N., Euring D., Cha J.Y., Lin Z., Lu M.Z., Huang L.J., Kim W.Y. (2021). Plant hormone-mediated regulation of heat tolerance in response to global climate change. Front. Plant Sci..

[16] Li M., Tan S., Tan G., Luo Y., Sun B., Zhang Y., Chen Q., Wang Y., Zhang F., Zhang Y. (2020). Transcriptome Analysis Reveals Important Transcription Factor Families and Reproductive Biological Processes of Flower Development in Celery (*Apium graveolens* L.). Agronomy.

[17] Zheng Y., Xiao J., Zheng K., Ma J., He M., Li J., Li M. (2021). Transcriptome Profiling Reveals the Effects of Nitric Oxide on the Growth and Physiological Characteristics of Watermelon under Aluminum Stress. Genes.

[18] Callwood J., Melmaiee K., Kulkarni K.P., Vennapusa A.R., Aicha D., Moore M., Vorsa N., Natarajan P., Reddy U.K., Elavarthi S. (2021). Differential morpho-physiological and transcriptomic responses to heat stress in two blueberry species. Int. J. Mol. Sci..

[19] Hardeland R., Cardinali D.P., Srinivasan V., Spence D.W., Brown G.M., Pandi-Perumal S.R. (2011). Melatonin—A pleiotropic, orchestrating regulator molecule. Prog. Neurobiol..

[20] Reiter R.J., Rosales-Corral S.A., Tan D.X., Acuna-Castroviejo D., Qin L.L., Yang S.F., Xu K.X. (2017). Melatonin, a full service anti-cancer agent: Inhibition of initiation, progression and metastasis. Int. J. Mol. Sci..

[21] Xiao S., Liu L.T., Wang H., Li D.X., Bai Z.Y., Zhang Y.J., Sun H.C., Zhang K., Li C.D. (2019). Exogenous melatonin accelerates seed germination in cotton (*Gossypium hirsutum* L.). PLoS ONE.

[22] Erdal S. (2019). Melatonin promotes plant growth by maintaining integration and coordination between carbon and nitrogen metabolisms. Plant Cell Rep..

[23] Sun Q.Q., Zhang N., Wang J.F., Zhang H.J., Li D.B., Shi J., Li R., Weeda S., Zhao B., Ren S.X. (2015). Melatonin promotes ripening and improves quality of tomato fruit during postharvest life. J. Exp. Bot..

[24] Lin X.X., Wang L., Hou Y.Y., Zheng Y.H., Jin P. (2020). A combination of melatonin and ethanol treatment improves postharvest quality in bitter melon fruit. Foods.

[25] Poeggeler B., Saarela S., Reiter R.J., Tan D.X., Chen L.D., Manchester L.C., Barlow-Walden L.R. (1994). Melatonin—A highly potent endogenous radical scavenger and electron donor: New aspects of the oxidation chemistry of this indole accessed in vitro. Ann. N. Y. Acad. Sci..

[26] Bawa G., Feng L.Y., Shi J.Y., Chen G.P., Cheng Y.J., Luo J., Wu W.S., Ngoke B., Cheng P., Tang Z.Q. (2020). Evidence that melatonin promotes soybean seedlings growth from low-temperature stress by mediating plant mineral elements and genes involved in the antioxidant pathway. Funct. Plant Biol..

[27] Li C., Tan D.X., Liang D., Chang C., Jia D.F., Ma F.W. (2015). Melatonin mediates the regulation of ABA metabolism, free-radical scavenging, and stomatal behaviour in two Malus species under drought stress. J. Exp. Bot..

[28] Shi H.T., Tan D.X., Reiter R.J., Ye T.T., Yang F., Chan Z.L. (2015). Melatonin induces class A1 heat-shock factors (HSFA1s) and their possible involvement of thermotolerance in *Arabidopsis*. J. Pineal Res..

[29] Fleta-Soriano E., Díaz L., Bonet E., Munné-Bosch S. (2017). Melatonin may exert a protective role against drought stress in maize. J. Agron. Crop Sci..

[30] Gao W.Y., Feng Z., Bai Q.Q., He J.J., Wang Y.J. (2019). Melatonin-mediated regulation of growth and antioxidant capacity in salt-tolerant naked oat under salt stress. Int. J. Mol. Sci..

[31] Amjadi Z., Namdjoyan S., Soorki A.A. (2021). Exogenous melatonin and salicylic acid alleviates cadmium toxicity in safflower (*Carthamus tinctorius* L.) seedlings. Ecotoxicology.

[32] Manafi H., Baninasab B., Gholami M., Talebi M., Khanizadeh S. (2022). Exogenous melatonin alleviates heatinduced oxidative damage in strawberry (*Fragaria* × *ananassa* Duch. cv. Ventana) plant. J. Plant. Growth Regul..

[33] Li M.Y., Feng K., Hou X.L., Jiang Q., Xu Z.S., Wang G.L., Liu J.X., Wang F., Xiong A.S. (2020). The genome sequence of celery (*Apium graveolens* L.), an important leaf vegetable crop rich in apigenin in the Apiaceae family. Hortic. Res..

[34] Li M., Li J., Zhang R., Lin Y., Xiong A., Tan G., Luo Y., Zhang Y., Chen Q., Wang Y. (2022). Combined Analysis of the Metabolome and Transcriptome to Explore Heat Stress Responses and Adaptation Mechanisms in Celery (*Apium graveolens* L.). Int. J. Mol. Sci..

[35] Corey Lesk C., Rowhani P., Ramankutty N. (2016). Influence of extreme weather disasters on global crop production. Nature.

[36] Zhang N., Sun Q.Q., Zhang H.J., Cao Y.Y., Weeda S., Ren S.X., Guo Y.D. (2015). Roles of melatonin in abiotic stress resistance in plants. J. Exp. Bot..

[37] Khan M.N., Zhang J., Luo T., Liu J.H., Rizwan M., Fahad S., Xu Z.H., Hu L.Y. (2019). Seed priming with melatonin coping drought stress in rapeseed by regulating reactive oxygen species detoxification: Antioxidant defense system, osmotic adjustment, stomatal traits and chloroplast ultrastructure perseveration. Ind. Crops Prod..

[38] Shen H.F., Zhao B., Xu J.J. (2016). Relationship between leaf anatomy and heat tolerance of 15 rhododendron cultivars. Chin. J. Appl. Ecol..

[39] Zhang J., Shi Y., Zhang X.Z., Du H.M., Xu B., Huang B.R. (2017). Melatonin suppression of heat-induced leaf senescence involves changes in abscisic acid and cytokinin biosynthesis and signaling pathways in perennial ryegrass (*Lolium perenne* L.). Environ Exp. Bot..

[40] Moradpour M., Abdullah S.N.A., Namasivayam P. (2021). The impact of heat stress on morpho-physiological response and expression of specific genes in the heat stress-responsive transcriptional tegulatory network in *Brassica oleracea*. Plants.

[41] Zhang J., Jiang X.D., Li T.L., Cao X.J. (2014). Photosynthesis and ultrastructure of photosynthetic apparatus in tomato leaves under elevated temperature. Photosynthetica.

[42] Cui G.B., Zhao X.X., Liu S.D., Sun F.L., Zhang C., Xi Y.J. (2017). Beneficial effects of melatonin in overcoming drought stress in wheat seedlings. Plant Physiol. Biochem..

[43] Huang B., Chen Y.E., Zhao Y.Q., Ding C.B., Liao J.Q., Hu C., Zhou L.J., Zhang Z.W., Yuan S., Yuan M. (2019). Exogenous melatonin alleviates oxidative damages and protects photosystem II in maize seedlings under drought stress. Front. Plant Sci..

[44] Yin L.H., Wang P., Li M.J., Ke X.W., Li C.Y., Liang D., Wu S., Ma X.L., Li C., Zou Y.J. (2013). Exogenous melatonin improves *Malus* resistance to marssonina apple blotch. J. Pineal Res..

[45] Zhang Z.K., Wang T., Liu G.S., Hu M.J., Yun Z., Duan X.W., Cai K., Jiang G.X. (2021). Inhibition of downy blight and enhancement of resistance in litchi fruit by postharvest application of melatonin. Food Chem..

[46] Zhang H.X., Jin J.H., He Y.M., Lu B.Y., Li D.W., Chai W.G., Khan A., Gong Z.H. (2016). Genome-wide identification and analysis of the SBP-Box family genes under phytophthora capsici stress in pepper (*Capsicum annuum* L.). Front. Plant Sci..

[47] Wang P.J., Chen D., Zheng Y.C., Jin S., Yang J.F., Ye N.X. (2018). Identification and expression analyses of SBP-Box genes reveal their involvement in abiotic stress and hormone response in tea plant (*Camellia sinensis*). Int. J. Mol. Sci..

[48] Feng X., Yu Q., Zeng J.B., He X.Y., Liu W.X. (2022). Genome-wide identification and characterization of GATA family genes in wheat. BMC Plant Biol..

[49] Babbar R., Karpinska B., Grover A., Foyer C.H. (2021). Heat-induced oxidation of the nuclei and cytosol. Front. Plant Sci..

[50] Martinez V., Nieves-Cordones M., Lopez-Delacalle M., Rodenas R., Mestre T.C., Garcia-Sanchez F., Rubio F., Nortes P.A., Mittler R., Rivero R.M. (2018). Tolerance to stress combination in tomato plants: New insights in the protective role of melatonin. Molecules.

[51] Zhao G., Yu X.L., Lou W., Wei S.Q., Wang R., Wan Q., Shen W.B. (2019). Transgenic Arabidopsis overexpressing *MsSNAT* enhances salt tolerance via the increase in autophagy, and the reestablishment of redox and ion homeostasis. Environ. Exp. Bot..

[52] Shanga F.Z., Liu R.L., Wu W.J., Han Y.C., Fang X.J., Chen H.J., Haiyan Gao H.Y. (2021). Effects of melatonin on the components, quality and antioxidant activities of blueberry fruits. LWT.

[53] Zhang X., St Leger R.J., Fang W.G. (2017). Pyruvate accumulation is the first line of cell defense against heat stress in a fungus. mBio.

[54] Benjamini Y., Hochberg Y. (1995). Controlling the false discovery rate: A practical and powerful approach to multiple testing. J. R. Stat. Soc. Ser. B Methodol..

