# Supplementary material for: Comparative Physiological and Transcriptomic Analyses of Improved Heat Stress Tolerance in Celery (Apium Graveolens L.) Caused by Exogenous Melatonin
Source: Int J Mol Sci. 2022 Sep 27;23(19):11382. doi: 10.3390/ijms231911382 (PMC9569527; doi:10.3390/ijms231911382)
Supplement: Supplementary file 1 [file ijms-23-11382-s001.zip › Figure S1.pdf]

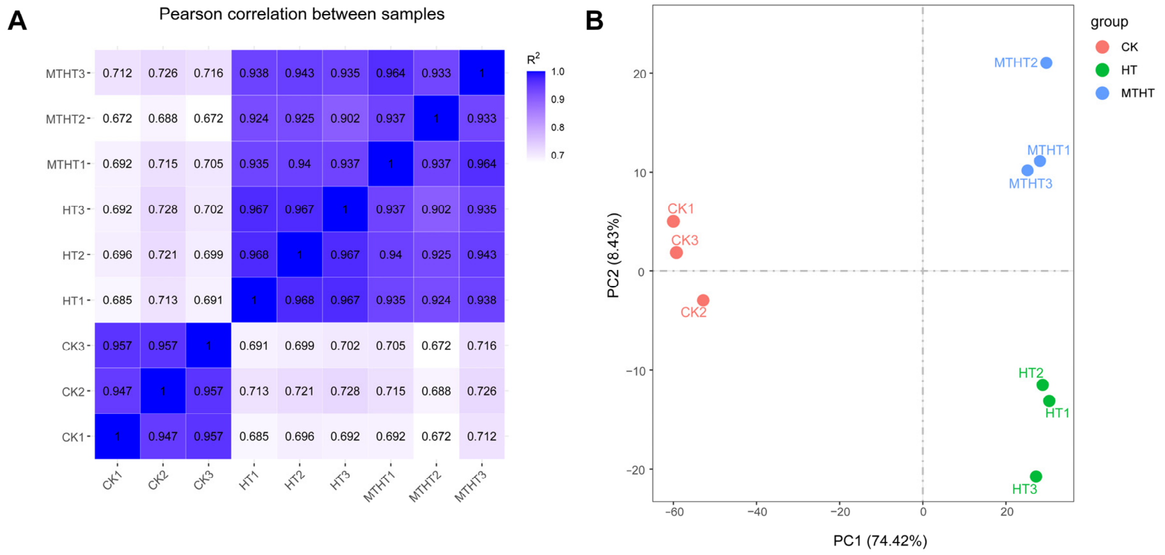

Figure S1. (**A**) Pearson coefficients (PCCs) of gene expression under different treatments (CK, HT, and MTHT) for three replicates presented. (**B**) PCA score plots of transcriptome data for three groups.
